# A Coordination-Driven Self-Assembly and NIR Photothermal Conversion Study of Organometallic Handcuffs

**DOI:** 10.3390/molecules28196826

**Published:** 2023-09-27

**Authors:** Xiaoyan Lu, Jing-Jing Huang, Tian Chen, Jie Zheng, Ming Liu, Xin-Yi Wang, Yu-Xin Li, Xinkai Niu, Li-Long Dang

**Affiliations:** 1 College of Chemistry and Chemical Engineering, Luoyang Normal University, Luoyang 471934, China; 2 Luoyang Institute of Science and Technology, Luoyang 471023, China; 3 College of Materials and Chemical Engineering, China Three Gorges University, Yichang 443002, China; 4 College of Science, Shihezi University, Shihezi 832003, China

**Keywords:** coordination-driven self-assembly, half-sandwich fragment, organometallic handcuffs, photothermal conversion

## Abstract

Due to their fascinating topological structures and application prospects, coordination supramolecular complexes have continuously been studied by scientists. However, the controlled construction and property study of organometallic handcuffs remains a significant and challenging research subject in the area of supramolecular chemistry. Hence, a series of tetranuclear organometallic and heterometallic handcuffs bearing different size and metal types were rationally designed and successfully synthesized by utilizing a quadridentate pyridyl ligand (tetra-(3-pyridylphenyl)ethylene) based on three Cp*Rh (Cp* = *η*^5^-C_5_Me_5_) fragments bearing specific longitudinal dimensions and conjugated planes. These results were determined with single-crystal X-ray diffraction analysis technology, ESI-MS NMR spectroscopy, etc. Importantly, the photoquenching effect of Cp* groups and the discrepancy of intermolecular π–π stacking interactions between building block and half-sandwich fragments promote markedly different photothermal conversion results. These results will further push the synthesis of topological structures and the development of photothermal conversion materials.

## 1. Introduction

In recent decades, coordination supramolecular chemistry has made rapid progress and has become an important chemical field not only due to its exquisite topological structures but also for its dependence on promising application prospects in covering host–guest chemistry [1,2,3,4], biosimulation [5,6,7], fluorescence [8,9,10], photothermal response [11,12,13], etc. In the synthesis process of these complexes, the type of structure changes dramatically from simple to complicated topologies or from low to high order winding. After the efforts of previous scientists, the synthesis of initial macrocyclic compounds has evolved from [2] catenane compounds to Solomon, Borromean ring and [5] catenane structures, which have been well demonstrated by Sauvage, Stoddart, Fujita and Jin, etc. [14,15,16,17,18,19,20,21,22,23], marking the rapid development of the research field. At the same time, molecular knots and ravel have also experienced a rapid forward process. Studies from the original synthesis of trefoil knots have evolved to the construction of intricate knots and ravels in more than five crossings, including a pentafoil knot; +3_1_ #+3_1_ #+3_1_ composite knot; granny knot and 8_18_, 5_2_, 7_1_, and 9_1_ knots [24,25,26,27,28,29]. These results pushed synthetic chemistry to a more sophisticated level. In addition, some non-intercable ring-in-ring structures and Russian doll assemblies are also continuously constructed to fill the scope of these exquisite topologies. Accompanied by the pursuit of the synthesis of these structures, structural transformations were explored by guest-, solvent- and metal ion-induced effects plus some chemical reactivity, etc., making it possible to synthesize some difficult compounds.

Scientists are also examining the properties of and applications for these complicated compounds. Some studies of organometallic macrocycle [30,31,32,33], cage [34,35,36], catenanes on luminescence [37,38,39], host–guest chemistry [40,41,42], separation [43,44,45], chiral resolution and catalysis [46,47,48] have made good progress. Professor Han proposed a new strategy to synthesize various carbinyl macrocyclic compounds and acquired some [2+2] cycloaddition organic products [49,50,51]. Zhang group synthesized a series of Pt^2+^ and Pd^2+^ based metal–organic cage compounds and investigated their aggregation-induced luminescence properties [38,39]. All these studies have played a great role in promoting research in this field.

Among the compounds constructed, a synthesis and property study of molecular handcuff compounds have been less explored due to the spatial effect of the interior structure, which makes the design difficult. Therefore, achieving synthesis and performing the property study of molecular handcuffs is significantly challenging.

Half-sandwich fragments have shown obvious advantages in the synthesis of supramolecular compounds due to their good solubility, crystallinity and directional coordination features. A series of specific supramolecular structures have been successfully synthesized, such as Borromean rings, Solomon links, [2,3,4,5] catenanes, 4-ravel and various knots [12,15,17,18,19,21,31]. Therefore, choosing appropriate organic linkers and half-sandwich building blocks with specific sizes might enable the construction of molecular handcuffs. In addition, the fluorescence quenching effect of half-sandwich fragments and the different intermolecular π–π stacking interactions possibly induce strong photothermal conversion properties.

**Scheme 1:**
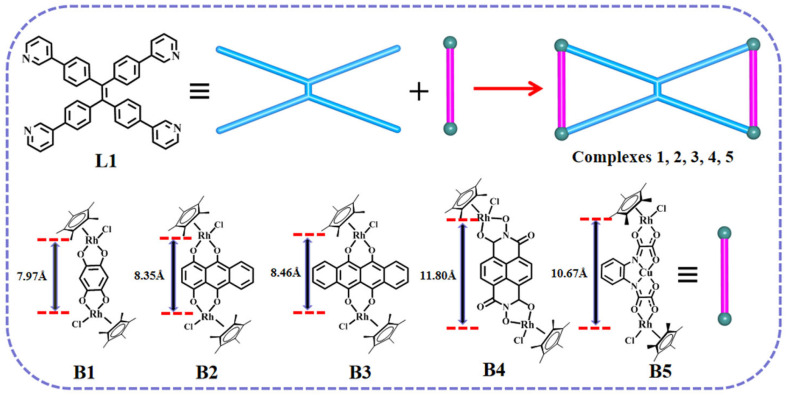
The designed ligand **L1** and building blocks **B1**, **B2, B3, B4** and **B5**.

**L1**, a tetradentate pyridine ligand, was chosen. In it, the central olefinic bond unit can keep 4 pyridine units in the same plane and generates a particular twist. The characteristics of a 3-pyridine unit could facilitate the coordination of 2 pyridine sites in the same ligand with half-sandwich metal ions, providing the conditions for the formation of molecular handcuffs. Here, a series of organometallic molecular handcuffs based on **B1**–**B5** building blocks bearing different lateral width and longitudinal dimensions were successfully constructed (Scheme 1). Structures were clearly confirmed by single crystal X-ray diffraction and NMR spectra. Additionally, photothermal conversion experiments have shown that these structures have different photothermal conversion properties due to differences in the size and conjugation effect of the building blocks **B1**, **B2, B3** and **B4**. Compound **4** exhibited a remarkable photothermal conversion performance.

## 2. Results and Discussion

### 2.1. The Synthesis of Organometallic Handcuffs **1**, **2**, **3**

**Scheme 2:**
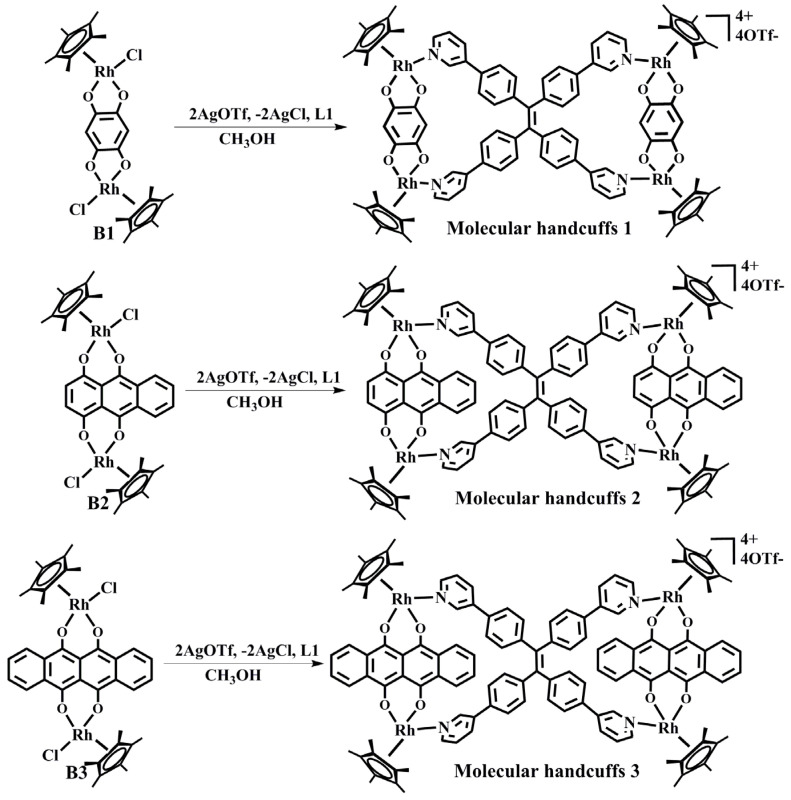
The synthesis of molecular handcuff compounds (**1**, **2** and **3**).

The organic ligand **L1** and the three binuclear complexes **B1, B2** and **B3** were synthesized based on a previously reported method in the literature. The molecular handcuffs **1**, **2** and **3** were obtained by the reactions between ligand **L1** and **B1, B2** or **B3**, as shown in Scheme 2. **B1**, **B2** or **B3** was first reacted with two equivalents of silver trifluoromethanesulfonate (AgOTf) under dark conditions. Then, silver chloride was removed through centrifugation so that rhodium salt could be connected to the bridging donor ligand **L1**. Subsequently, the **L1** ligand was added into the solution above, and products **1**, **2** and **3** were extracted with methanol/isopropyl ether with yields of 85.7%, 84.8% and 83.4%, respectively. At room temperature, isopropyl ether was slowly diffused into a solution of complex **1** in methanol, and over a period of two days, single crystals of complex **1** were obtained.

### 2.2. The Self-Assembly and Structural Analysis of Molecular Handcuffs **1**

**Figure 1:**
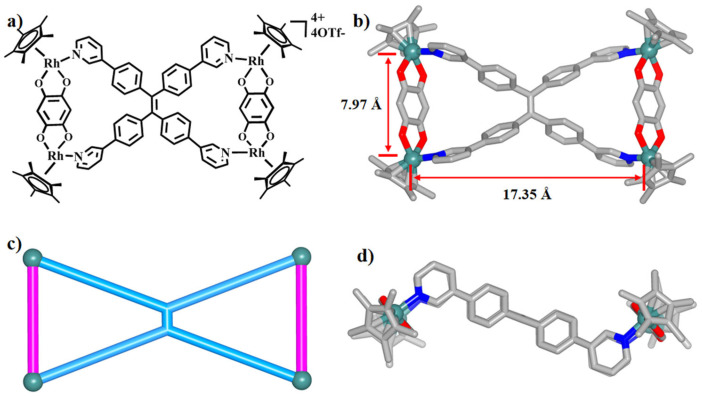
(**a**) The chemical structure of molecular handcuffs **1;** (**b**) the solid-state structure of **1**, showing the Rh–Rh nonbonding distances; (**c**) a simplified topological representation of the structure of **1**; (**d**) a top view of the structure of 1. All hydrogen atoms were omitted for clarity (N, blue; C, gray; Rh, Aqua; Cl, green).

Single-crystal X-ray analysis and an NMR spectrum confirmed the structure of complexes **1** as shown in Figure 1. The two Rh^III^ molecular clips in **B1** are linked to four pyridyl sites of one ligand **L1**, generating two rings connected by a central olefinic bond, forming a very interesting handcuffed complex **1**, which exhibits short and long Rh–Rh nonbonding distances of 7.97 and 17.35 Å. Viewed from the side, the two molecular clips **E1** are placed in a trans configuration, generating a Z-shaped structure. Furthermore, the stacking pattern analysis revealed no interactions between the molecules, suggesting that individual discrete molecules can be stable.

**Figure 2:**
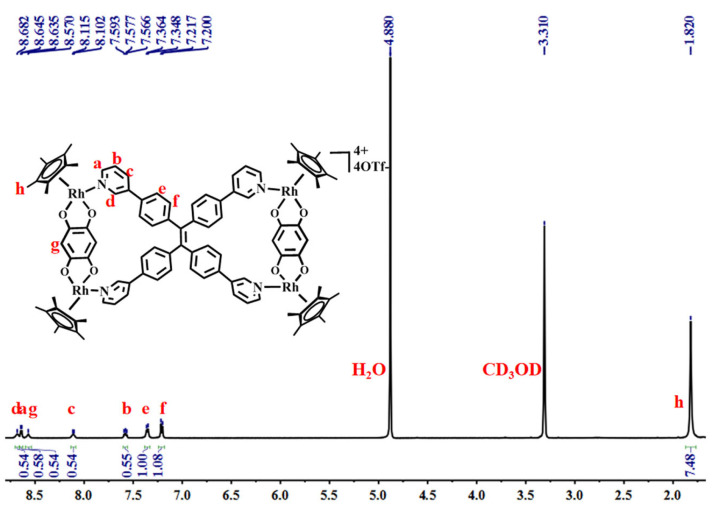
The ^1^H NMR (500 MHz, CD_3_OD, ppm) for **1** (7.0 mM, with respect to Cp*Rh).

**Figure 3:**
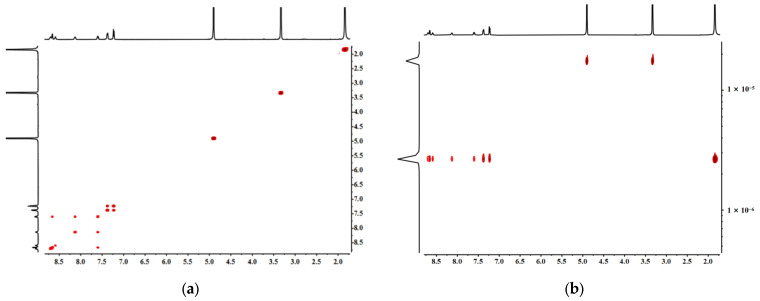
(**a**) The ^1^H-^1^H COSY NMR (500 MHz, CD_3_OD, ppm) for **1**; (**b**) The ^1^H-^1^H DOSY NMR (500 MHz, CD_3_OD, ppm) for **1** (2.74 × 10^−6^ cm^2^s^−1^) (7.0 mM, with respect to Cp*Rh).

**Figure 4:**
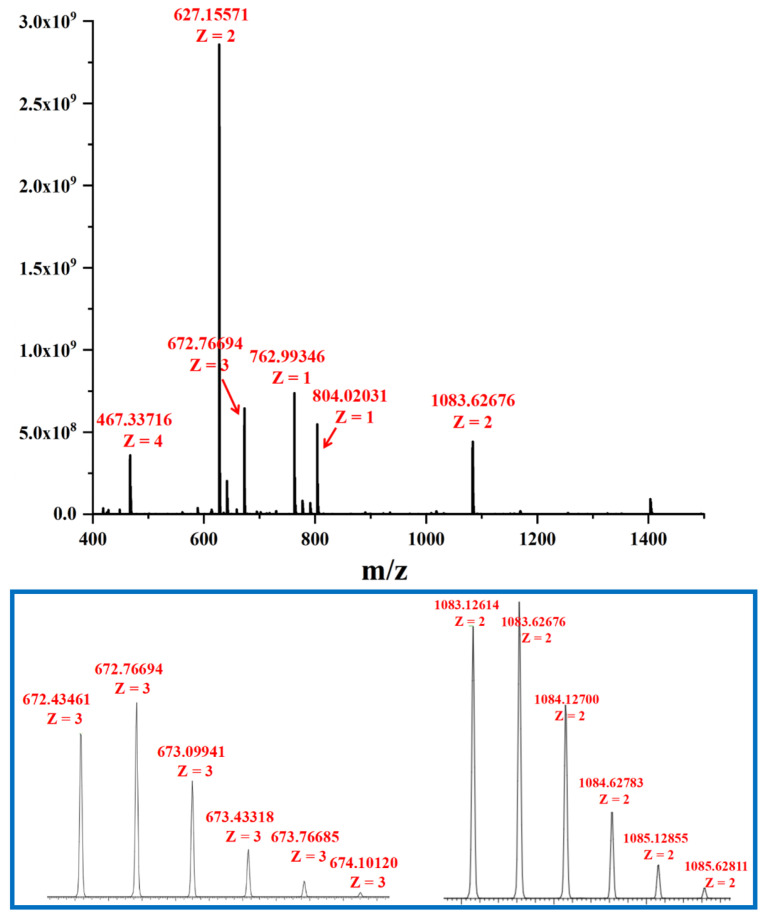
The full ESI-TOF-MS spectrum of complex **1**; 2^+^ and 3^+^ mass peaks of **1**.

Additionally, the presence of molecular handcuffs **1** under solution conditions were also demonstrated by the ^1^H NMR spectrum (Figure 2), accompanied by the combination of ^1^H-^1^H COSY NMR and ^1^H-^1^H DOSY NMR spectra (Figure 3a,b). Two obvious coupled double peaks could be observed at 8.64 and 8.11 ppm in complex **1**, and it displayed an obvious coupling interaction with the triplet at 7.58 ppm, which belonged to the a, b, c positions of the meta-position pyridine group of **L1**. Two phenyl proton signals—e, f—could be found at 7.35, 7.21 ppm and 8.68 ppm. Additionally, two strong singlets in the aromatic region could be found at 8.68 and 8.57 ppm, which belonged to the pyridyl proton and benzoquinone proton building block **E1**. In addition, the signal of Cp* group was at 1.82 ppm (Figure 2). The ^1^H DOSY NMR spectrum of **1** demonstrated that signals for the aromatic and Cp* units displayed a single diffusion coefficient (2.74 × 10^−6^ cm^2^ s^−1^), further determining the stable presence of **1** (Figure 3b). In addition to NMR spectroscopic data, the presence of complex **1** was also determined by ESI-MS: [1-2OTf^−^]^2+^ (*m*/*z* = 1083.12) and [1-3OTf^−^]^3+^ (*m*/*z* = 672.43) (Figure 4).

### 2.3. The Self-Assembly and Structural Analysis of Molecular Handcuffs **2** and **3**

**Figure 5:**
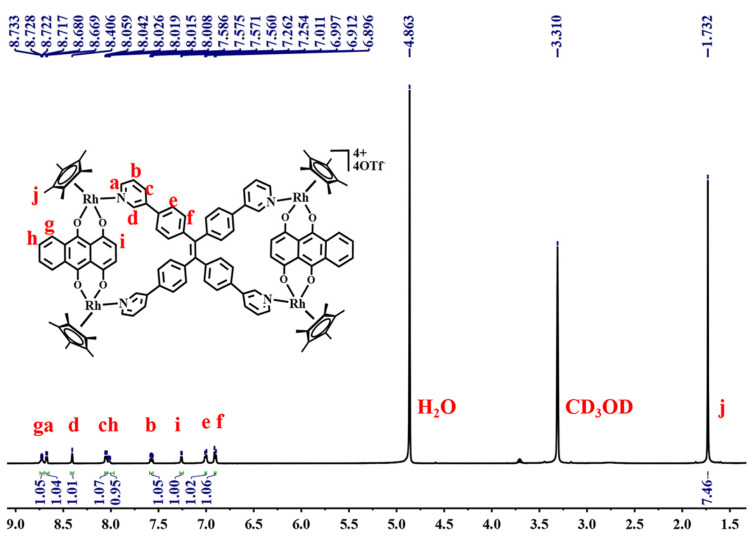
The ^1^H NMR (500 MHz, CD_3_OD, ppm) for **2** (12.0 mM, with respect to Cp*Rh).

**Figure 6:**
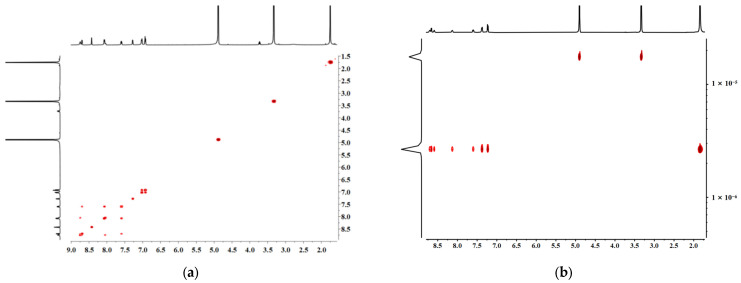
(**a**) The ^1^H-^1^H COSY NMR (500 MHz, CD_3_OD, ppm) for **2** and (**b**) the ^1^H-^1^H DOSY NMR (500 MHz, CD_3_OD, ppm) for **2** (2.88 × 10^−6^ cm^2^s^−1^) (12.0 mM, with respect to Cp*Rh).

The molecular handcuff structure **1** prompts us to explore whether increasing the width of a Cp*-based building block can induce the difference in topological structure. Therefore, building blocks **B2** and **B3** were individually introduced into the system to carry out a reaction with ligand **L1**. Once the two reaction processes were completed, the NMR exploration experiments of complexes **2** and **3** were performed. The ^1^H NMR of complex **1** exhibited a clear set of proton signals (Figure 5), which were analyzed through the combination with the two-dimensional spectra of ^1^H-^1^H COSY NMR and ^1^H-^1^H DOSY NMR (Figure 6a,b). Two doublet signals at 8.68 ppm and 8.05 ppm were clearly observed, which had an obvious coupling effect with the same triplet at 7.57 ppm. Thus, the three signals belonged to the pyridinyl protons of ligand **L1**. A pair of coupled doublets at 7.01 ppm and a singlet in 6.90 ppm were due to the pyridinyl and phenyl protons of **L1**. In addition, a doublet at 7.26 ppm without coupling with other protons and two coupling multiple peaks at 8.73 and 8.02 ppm were due to the phenyl protons of the **E2** building units. The chemical shift of the Cp* proton was at 1.73 ppm. A single diffusion coefficient from the ^1^H-^1^H DOSY NMR spectrum (2.88 × 10^−6^ cm^2^s^−1^) further demonstrated the presence of complex **2** (Figure 6b). Likewise, the ^1^H NMR of complex **3** was analyzed, also showing a set of simple and clear proton signals. Most proton conditions were similar to those in compounds **1** and **2,** demonstrating the formation of molecular handcuffs **3** (Figure 1, Figure 2 and Figure 3). This study showed that increasing the width did not change the structural types of molecular handcuffs. 

### 2.4. The Self-Assembly of Molecular Handcuffs **4** and Heterometallic Handcuffs **5**

**Scheme 3:**
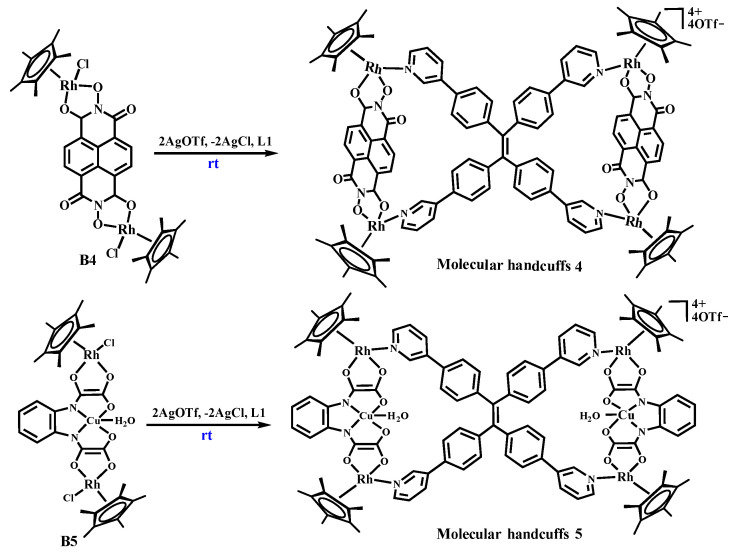
The synthesis of handcuffs **4** and heterometallic handcuffs **5**.

Next, to explore the effect of the size of the building blocks on the structural synthesis, two longer building blocks **B4** and a heterometallic [Cp*_2_Rh_2_L^Cu^]Cl_2_ (L^Cu^ = [Cu(opba)]^2−^ [opba = o-phenylenebis(oxamato)] (**B5**) were deliberately selected to synthesize a bigger organometallic assembly, in which the Rh–Rh nonbonding distances were 11.80 and 10.67 Å. Through the self-assembly of ligand **L1** and building blocks **B4** and **B5**, two new gray and green centrosymmetric complexes **4** and **5** were obtained in the yields of 85.8% and 83.6% (Scheme 3). Despite various attempts, we did not obtain a single crystal structure of complex **4**. Fortunately, the structure of complex **5** was ensured by single crystal X-ray diffraction.

**Figure 7:**
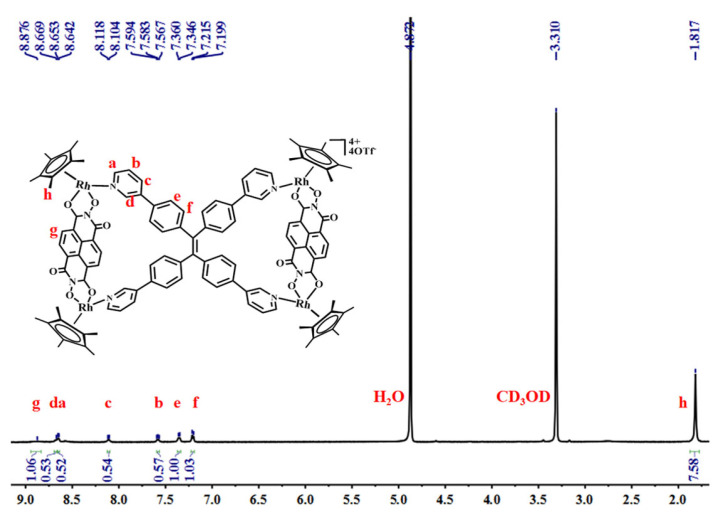
The ^1^H NMR (500 MHz, CD_3_OD, ppm) for **2** (7.0 mM, with respect to Cp*Rh).

**Figure 8:**
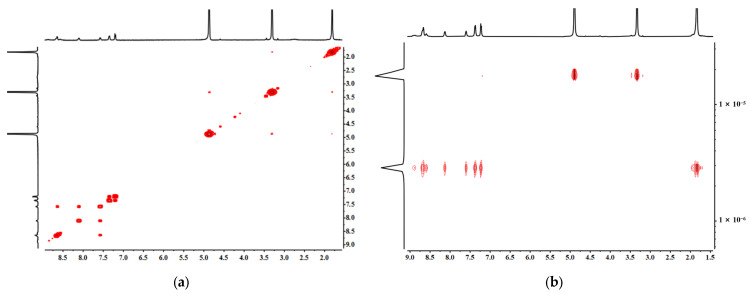
(**a**) The ^1^H-^1^H COSY NMR (500 MHz, CD_3_OD, ppm) for **4** and (**b**) the ^1^H-^1^H DOSY NMR (500 MHz, CD_3_OD, ppm) for **4** (2.34 × 10^−6^ cm^2^s^−1^) (7.0 mM, with respect to Cp*Rh).

Complex 4 was dissolved in deuterated methanol to explore its solution behavior. The ^1^H NMR of complex **4** displayed a set of clear proton signals (Figure 7), which were determined by the utilization of the two-dimensional spectra of ^1^H-^1^H COSY NMR and ^1^H-^1^H DOSY NMR (Figure 8a,b). A triplet at 7.59 ppm has obvious coupling interactions with two doublets at 8.65 and 8.10 ppm, which is consistent with the three adjacent proton environments of the meta-position pyridine group of **L1**; these protons could be attributed to the a, b, c positions of the pyridine unit. In addition, two doublets at 7.34 and 7.22 ppm with coupling interactions belonged to e, f of the phenyl unit. Additionally, two strong singlets in the aromatic region could be found at 8.68 and 8.57 ppm. Furthermore, the signals of the NDI unit and Cp* group in building block B4 could be located at 8.88 and 1.82 ppm (Figure 6). The ^1^H DOSY NMR spectrum of **4** obviously exhibited that these signals for the aromatic and Cp* units displayed a single diffusion coefficient (2.34 × 10^−6^ cm^2^s^−1^), further determining the stable presence of **4** (Figure 8b). 

**Figure 9:**
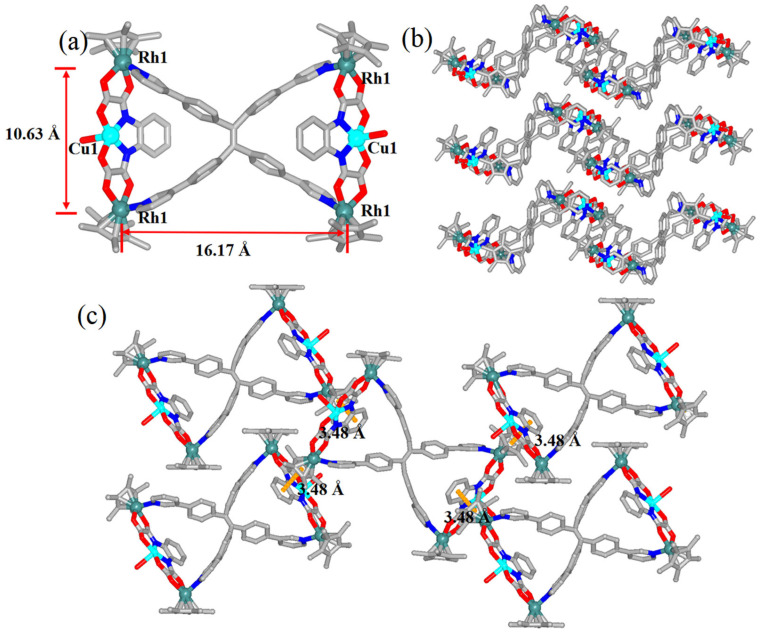
(**a**) The molecular structure of heterometallic handcuffs **5**. (**b**) A side view of heterometallic handcuffs 4. (**c**) A top view of heterometallic handcuffs 4 (π–π stacking interactions: 3.48 Å). OTf^−^ and solvent molecules outside the framework and hydrogen atoms are omitted (O, red; N, blue; C, gray; H, pink; Rh, Aqua; Cu, cyan).

Moreover, single crystals suitable for X-ray structure determination were obtained via slow vapor diffusion of isopropyl ether into a methanol solution of **5** at room temperature, displaying the acquisition of interesting heterometallic handcuffs, which included two metal ions, rhodium ions and copper ions. The single crystal structure showed that the distance between the two Rh–Rh that were coordinated with the pyridinyl group was about 11.80 and 10.67 A, respectively. The longest distance of Rh–Rh was 16.17 Å, which is similar to the distances of complexes **1** and **2**. Careful observation revealed that an obvious accumulation effect (3.48 Å) between Cp* and the phenyl group could be observed, reflecting strong intermolecular interactions. These interactions caused the formation of two-dimensional layered structures. (Figure 9). 

### 2.5. Photothermal Conversion Studies

**Figure 10:**
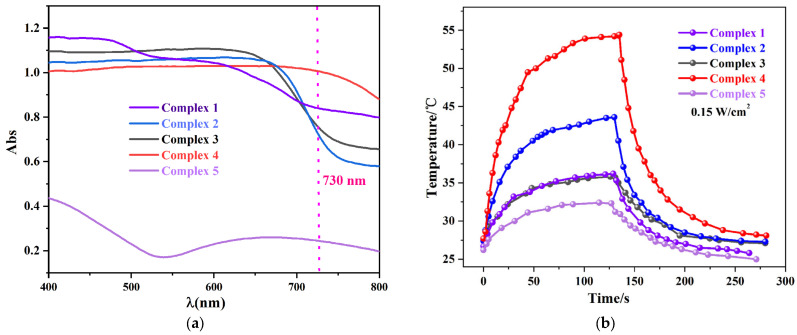
(**a**) The UV–Vis absorption spectrum of complexes **1**, **2**, **3**, **4** and **5** (400–800 nm); and (**b**) the NIR photothermal conversion curves of metallarectangles **1**, **2**, **3**, **4** and **5**.

Photothermal materials are widely used in the fields of photothermal therapy, photothermal imaging and photothermal sensors [52,53]. Previous research showed that half-sandwich structures have a very strong light quenching effect. The dark appearance and near infrared absorption of these half-sandwich-based molecular handcuffs prompted us to investigate their photothermal conversion performance, which have also attracted more and more attention of scientists [54]. First, the solid UV-visible spectra of complexes **1**, **2**, **3**, **4** and **5** were recorded (Figure 10a). Results showed a set of obvious near infrared absorption: 0.84, 0.70, 0.74, 1.0 and 0.22 at 730 nm for complexes **1**, **2**, **3**, **4** and **5**, respectively. Analyzing these absorption values carefully, we could find that complexes **4** and **5** showed the strongest and weakest near infrared absorption, which are related to their conjugation effect and copper ion coordination characteristics. This might have resulted in the strongest and weakest photothermal conversion effect. Meanwhile, a similar absorption value of complexes **1**, **2**, **3** could be attributed to their close conjugation effect. These near infrared absorption values could generate a corresponding NIR photothermal conversion ability. 

Subsequently, the NIR photothermal conversion studies for complexes **1**, **2**, **3**, **4** and **5** were separately carried out. And the photothermal conversion curves of these complexes showed a specific heating process. The temperature of complex **1** under 0.6 W/cm^2^ laser irradiation at a 730 nm wavelength displayed a significant increase at 9.5 °C (from 26.7 to 36.2 °C, Figure 10b). Meanwhile, the observed temperature changes for complexes **2** (from 27.4 to 43.6 °C) and **3** (from 27.4 to 35.8 °C) are similar to that recorded for complex **1**. Importantly, an obvious stronger photothermal conversion could be observed for molecular handcuffs **4**. Thus, a temperature increase of 26.7 °C (from 27.7 to 54.4 °C) in 230 s under a power irradiation (0.15 W/cm^2^, Figure 10b), which could be due to the suitable conjugation effect of building block **B4**, resulted in the strongest near infrared (NIR) absorption (1.0) at 730 nm and intermolecular π–π stacking interactions, leading to a good NIR photothermal conversion effect. Nevertheless, just as we expected, the weak near infrared absorption (0.22) at 730 nm and poor intermolecular π–π stacking interactions in complex **5** resulted in a very small temperature change of about 6.4 °C (from 26.2 °C to 32.4 °C), which was consistent with our initial speculation.

**Figure 11:**
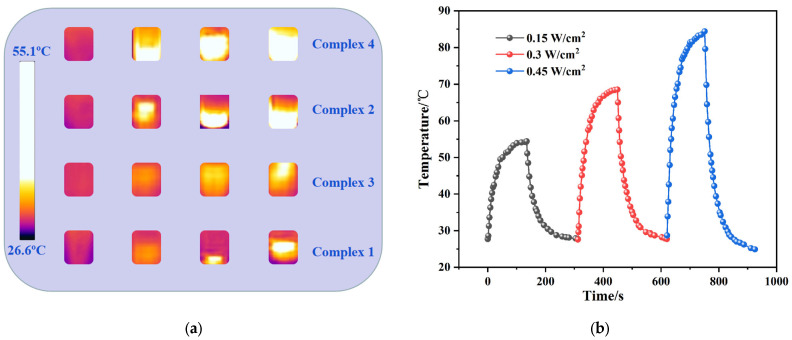
(**a**) NIR thermal images of metallarectangles **1**, **2**, **3**, and **4** in the spectrophotometer cell (1 × 1 × 5 cm) under 730 nm laser irradiation and (**b**) NIR photothermal conversion curves of 4 under 0.15, 0.3, 0.45 W/cm^2^ laser irradiation at 730 nm.

Due to the excellent photothermal conversion performance of complex **4** at the laser power per unit area of 0.15 W/cm^2^ at 730 nm, we continued to study the photothermal conversion ability under different laser power per unit areas: 0.15, 0.3, 0.45 W/cm^2^ at 730 nm (Figure 11a). Results showed that accompanied by the increase in laser power per unit area from 0.15 to 0.45 W/cm^2^, temperature change increased accordingly (Figure 11b). Specifically, when the power per unit area was 0.45 W/cm^2^, the temperature change of complex **4** could reach 55.7 °C (from 28.7 °C to 84.4 °C), suggesting that in a special near infrared light region, the increase in laser power per unit area could result in the enhancing of the photothermal conversion performance.

## 3. Experiment

### Materials

All reagents and solvents were commercially purchased and used without further purification. The starting materials **B1**, **B2**, **B3**, **B4** and [Cp*_2_Rh_2_(L^Cu^)]Cl_2_ (**B5**) were synthesized based on a previously reported method in the literature. NMR spectra were taken at room temperature using Bruker AVANCE I 500 spectrometers. Elemental analyses were collected on an Elementar Vario EL III analyzer. 

## 4. Conclusions

In summary, a series of organometallic molecular handcuffs bearing different sizes and species of metal atoms were successfully acquired through the self-assembly of quadridentate pyridyl ligand **L1** and the five Cp*Rh based building blocks **B1**, **B2**, **B3**, **B4** and **B5**. The change of the width and length of building blocks did not cause the change of the type of molecular handcuffs. And, these complexes were characterized by single crystal X-ray diffraction analysis, NMR spectra and UV–Vis spectra. Interestingly, the photothermal conversion studies for these complexes are carefully performed, reflecting different photothermal conversion capacities. Discrepancy in photothermal conversion comes from the different conjugative effects of building blocks **B1**–**B5**. A strong conjugation effect and π–π stacking interactions induced good photothermal conversion results. We believe that our results may be useful for constructing various Cp*-based photothermal conversion materials and promoting the rapid development of fine materials and smart materials. 

## Data Availability

Not applicable.
